# Triaging informative *cis*-regulatory elements for the combinatorial control of temporal gene expression during *Plasmodium falciparum* intraerythrocytic development

**DOI:** 10.1186/s13071-015-0701-0

**Published:** 2015-02-05

**Authors:** Karen Russell, Richard Emes, Paul Horrocks

**Affiliations:** Institute for Science and Technology in Medicine, Keele University, Staffordshire, ST5 5BG UK; School of Veterinary Medicine and Science, University of Nottingham, Leicestershire, LE12 5RD UK; Advanced Data Analysis Centre, University of Nottingham, Leicestershire, LE12 5RD UK

**Keywords:** AP2 transcription factor, Bioinformatics, *Cis*-acting DNA motifs, Combinatorial control, Finding informative regulatory elements, Malaria, Stage-specific expression

## Abstract

**Background:**

Over 2700 genes are subject to stage-specific regulation during the intraerythrocytic development of the human malaria parasite *Plasmodium falciparum*. Bioinformatic analyses have identified a large number of over-represented motifs in the 5′ flanking regions of these genes that may act as *cis*-acting factors in the promoter-based control of temporal expression. Triaging these lists to provide candidates most likely to play a role in regulating temporal expression is challenging, but important if we are to effectively design *in vitro* studies to validate this role.

**Methods:**

We report here the application of a repeated search of variations of 5′ flanking sequences from *P. falciparum* using the Finding Informative Regulatory Elements (FIRE) algorithm.

**Results:**

Our approach repeatedly found a short-list of high scoring DNA motifs, for which cognate specific transcription factors were available, that appear to be typically associated with upregulation of mRNA accumulation during the first half of intraerythrocytic development.

**Conclusions:**

We propose these *cis-trans* interactions may provide a combinatorial promoter-based control of gene expression to complement more global mechanisms of gene regulation that can account for temporal control during the second half of intraerythrocytic development.

**Electronic supplementary material:**

The online version of this article (doi:10.1186/s13071-015-0701-0) contains supplementary material, which is available to authorized users.

## Background

The human malarial parasite *Plasmodium falciparum* adopts numerous morphologically distinct forms as it completes its complex life cycle in the human host and mosquito vector. As the parasite invades, colonises and multiplies within these diverse host environments a complex programme of developmentally-linked gene expression, utilising a diverse range of molecular mechanisms to exert control, has been described; for reviews see [1-3]. These are perhaps best exemplified during asexual intraerythrocytic development, where morphological transition from the newly invaded ring form progresses over a 48 hour period, through trophozoites and schizonts, to produce merozoites ready to reinitiate invasion in a new host erythrocyte. Over this 48 hr period, a well-defined cascade of peak mRNA steady-state accumulation has been described for some 50% of the parasite’s genome, with temporally- and functionally-linked clusters of genes being expressed in time to meet their biological demand [4-7].

With little apparent inter-strain variation in mRNA profiles during intraerythrocytic development, and minimal changes resulting from drug perturbations, this transcriptional cascade has been described as “hard-wired” [7-11]. Analyses of the molecular mechanisms that govern this developmentally-linked gene expression suggest that this “hard-wiring” is likely the result of globally-acting regulatory mechanisms, specifically; stage-specific variations in nucleosome positioning, processivity of the RNA polymerase II complex and stage-specific variations in the stability of the mRNA transcript [12-19]. Hypotheses that considered regulation of stage-specific gene expression exerted at the level of individual promoters, through specific transcription factor biding to *cis*-regulatory DNA motifs, fell out of favour in the early 2000′s due to the apparent absence of transcription factors in the *P. falciparum* genome [1,20,21]. In 2008, however, a restricted number of specific transcription factors, sharing the apetela 2 (AP2) DNA binding motif, were found in *P. falciparum*, with homologues quickly identified throughout all apicomplexans, leading to their designation as ApiAP2 transcription factors [22-25]. ApiAP2 have subsequently been shown to be critical regulators in the regulation of gene expression throughout the *Plasmodium spp.* life cycle as well as potentially playing a role in the monoallelic expression of the PfEMP1 virulence protein family through modulation of the local chromatin environment [26-31]. In 2010, using protein binding arrays, the cognate *cis*-acting DNA motif for 24 of the 27 *P. falciparum* ApiAP2 were determined [32]. Interestingly, these DNA motifs are widely distributed within intergenic regions, with many intergenic regions sharing multiple ApiAP2 binding sites. Whilst this multiplicity of ApiAP2 binding sites may represent the means for a model of multifactorial control (a point that will be picked up later), whether all predicted DNA binding sites actually act as *cis*-regulatory sites remains to be addressed. In the absence of well-defined transcription start sites for *P. falciparum*, our inability to relate the position of a predicted ApiAP2 to this key transcriptional landmark hampers our efforts to design functional studies to explore their role in the control of transcription initiation.

*In silico* approaches have also been used to identify DNA motifs enriched within the flanking sequence of genes that share temporal peak mRNA profiles, function (utilising Gene Ontology terms) or share homologues in other *Plasmodium spp* [33-38]. Unfortunately, the catalogue of motifs predicted by each approach poorly overlap. Moreover, searches typically take an arbitrary length of flanking sequence for analysis. Our recent work exploring the size of flanking sequences in *P. falciparum*, highlight the challenge with such an arbitrary approach as we showed that the size of intergenic regions flanking a gene varies according to the nature of the transcriptional activity that takes place over this region [39]. In this same study we also predict that transcription start sites lie further upstream of the start of the open reading frame than has previously been suggested and thus, key information may have been missed in these studies.

Recognising the challenge in defining DNA motifs that are *most likely* playing a role in the promoter-based control of transcription initiation in the absence of transcription start site data, we established a programme of work to; i) identify high-scoring DNA motifs that are *repeatedly* linked with genes that share the same temporal profile of peak mRNA accumulation and ii) undertake a search for potential new DNA motifs that lie further upstream from regions of intergenic sequences explored to date. To carry out this study we utilised the Finding Informative Regulatory Elements (FIRE) algorithm to explore correlations between DNA motifs located in intergenic sequences upstream of genes that share the same temporal profile of steady-state mRNA levels [33].

## Methods

The source code and *P. falciparum* accessory files for the FIRE algorithm were obtained from the authors of the original FIRE study [33] and utilised on a PC operating a UNIX environment using the default sensitivity and stringency settings. These files are currently hosted, and freely available, online at https://tavazoielab.c2b2.columbia.edu/FIRE/. 5′ gene flanking sequences were obtained from a bespoke PERL script (intergenic.dist.2FASTA.pl available from https://sites.google.com/site/emesbioinformatics/group-software) using the *P. falciparum* General Feature Format (GFF) and genome sequence file downloaded from PlasmoDB5.5 (https://www.plasmoDB.org/plasmo). The intergenic.dist.2FASTA.pl program allows the user to specify the windows of 5′ flanking sequence to occur (-1000 to 0 and-1500 to-500 bp upstream of the start codon) and whether to capture sequences up to adjacent flanking genes if they fall within this window, or only when a full 1000 bp intergenic sequence can be captured. The FIRE output files for each search secured in separate folders. The FIRE motif heat maps and FIRE interaction heat maps resulting from the search of Groups A to D are attached in the Additional file 1. Analysis of the distribution of mutual information score(s) for the same motif discovered in one search (singleton) or multiple searches were performed using a Kruskall-Wallace one way analysis of variance with Dunn’s post-test (GraphPad Prism v5.1). WebLogos of DNA-binding specificities of all 27 members of the ApiAP2 protein family from *P. falciparum* along with their mRNA abundance profiles during intraerythrocytic development were sourced from the protein binding array study of Campbell *et al.* [32].

## Results and discussion

### Repeated discovery of DNA motifs associated with the temporal cascade of transcription during intraerythrocytic development

The Finding Informative Regulatory Elements (FIRE) algorithm discovers DNA motifs whose presence or absence in gene flanking sequences provides the most information about the expression profile of the associated flanking gene. For *P. falciparum*, peak mRNA accumulation data for some 2700 genes is available from a published temporal microarray study undertaken in 2 hr increments over the entire 48 hr of intraerythrocytic development [4]. These data provide a continuous expression profile that can be used to discover overrepresented DNA motifs in the flanking intergenic sequences of genes that share the same temporal profile of peak mRNA accumulation. Using such an approach, FIRE has previously been used to discover 21 DNA motifs in a search of 1000 bp of 5′ flanking sequence in *P. falciparum* [33]. We adopt here an approach that searches different permutations of a more recent annotation of *P. falciparum* gene 5′ flanking sequences to identify DNA motifs that are repeatedly discovered – thus offering an insight into their likelihood as *cis*-regulatory elements. Using our own recently published observations relating to the likely placement of transcription start sites between 600–1350 bp upstream of *P. falciparum* open reading frames [39], we also use an additional, but same sized, window to search further upstream than the original FIRE study to explore whether any potential new informative regulatory sites can be determined.

**Figure 1:**
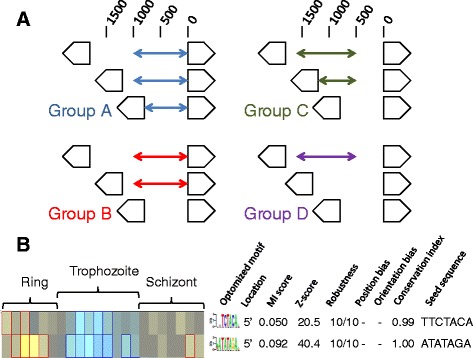
**Repeated FIRE searches of 5′ gene flanking regions in** ***P. falciparum*** **. (A)** Schematic representation of sequences used for the repeated FIRE analysis. The relative position of three hypothetical head-to-head orientated genes (pentagonal arrows) are illustrated with a scale for the intergenic distance (bp) shown above. In groups A and C, up to 1000 bp of intergenic sequence from either the start of the right-hand gene (group A) or from a position 500 bp upstream of the gene (group C) are secured. Where this 1000 bp window overlaps with the flanking left-hand gene, the sequences are truncated at the start of that gene. For groups B and D, the position of the window is the same as groups A and C, respectively. In this case only when all 1000 bp of sequence can be captured is this done. In this way, different, but related input sequence files can be subjected to a FIRE search. **(B)** Examples of FIRE motif heat-map output. The colour map illustrates over-representation (yellow) and under-representation (blue) of discovered DNA motifs in 5′ flanking regions of genes that share the same temporal profile of mRNA accumulation during intraerythrocytic development. The approximate morphological staging of these time points is illustrated above the colour map. To the right of the colour map the seed search sequence and the optimized motif (as a WebLogo) are shown along with qualitative and quantitative data reporting the score of the discovered motif, the reliability of the search and data relating to bias in position and orientation. These variables are explained in the main text. Full data for all groups are provided in Additional file 1.

We elected to use search windows of 1000 bp. Not only did this allow a comparison to the original FIRE study, but we have also recently shown that this distance represents approximately half of the median size of an intergenic space that contains two promoter regions in *P. falciparum* [39]. In total, four groups of 5′ flanking sequences (groups A to D) were secured for our analysis (Figure 1A). Group A most closely represents the sequences secured in the original FIRE report, i.e. 1000 bp of the most immediate flanking sequence. Based on our prediction that transcription start sites likely lie further upstream than considered in the original FIRE study, group C sequences were secured from a 1000 bp window located between 500 and 1500 bp upstream of each open reading frame. For both groups A and C, if the 1000 bp window overlapped with an adjacent open reading frame, the sequence captured was truncated to ensure only intergenic sequences were selected. Thus, two sets of sequences of up to 1000 bp for each gene were secured. Given our interest in repeatedly searching for the enrichment of the same DNA motif, two additional sets of upstream flanking sequences were secured. Whereas groups A and C captured up to 1000 bp of sequence, groups B and D secure the corresponding windows of 1000 bp sequence, respectively, but only when the entire 1000 bp sequence could be obtained. We hypothesised that those DNA motifs more likely associated with the control of stage-specific expression would be repeatedly identified in each of the groups, albeit with slightly different scores based on the different amount of sequences secured. Here, groups A to D consisted of 5579, 4300, 5297 and 3099 upstream flanking sequences, respectively.

FIRE analysis was performed on groups A to D, with 8–17 DNA motifs reported from each search. The algorithm produces a FIRE motif heat-map (see Figure 1B for example, see Additional file 1 for all files) for each search that provides a range of information for each DNA motif discovered. A colour-map is used to describe the correlation between either the over-representation (yellow) or under-representation (blue) of the DNA motif in genes that sharing the same peak mRNA accumulation profile (with the morphological stage representative of these timepoints indicated in Figure 1B). Correlations where this data is significantly over or under-represented (p < 0.05 after a Bonefori correction) are highlighted by bold red or blue surrounding lines, respectively. To the right of the heat map, the sequences of the seed motif for the search and the final optimized motif (as a WebLogo image) are shown alongside qualitative and quantitative evaluations of this DNA motif. Qualitatively, the location of the DNA motif in the 5′ flanking sequence is indicated for all motifs discovered here as well as any evidence of a positional or orientation bias following randomization trails. In the absence of well mapped transcription start sites in *P. falciparum* with which to correlate with these data, and the relatively few biases observed, no further analysis of these qualitative outcomes was performed here. The quantitative data reported includes; (i) mutual information, which indicates the extent of the association of the DNA motif with genes that share the same temporal profile of peak mRNA accumulation, (ii) the statistical significance (Z-score) of this association when compared to 10,000 randomizations of the input sequences, (iii) robustness of the association, i.e. how often the same motif is found in 10 separate jack-knife trials that remove one third of input sequences and (iv) the conservation index, indicates the shared presence of this motif in the flanking regions of orthologous genes in the murine malaria parasite *P. yoelii* (with indices of >0.95 considered significant).

Inspection of the lists of motifs identified in these searches reveal a total of 28 distinct DNA motifs (see Additional file 1). Of these, 14 had been previously described in the original FIRE study. The remaining 14 novel DNA motifs all share the common feature of each being discovered only once across groups A to D. A similar representation of singleton motif discovery in the original FIRE report can now be drawn by comparison to the searches performed here. Here, seven of the 21 motifs were not rediscovered in our analysis. Comparison of the mutual information scores between motifs discovered in two or more of the five groups (A to D and the original study) and those only discovered in a single search revealed a significantly lower score (one way analysis of variance with Dunns post-test, p < 0.05) in the singleton group. A second aspect of the search addressed whether searches for motifs in sequences located between 500 and 1500 bp upstream of the open reading frame would identify new motifs. Only four motifs were uniquely discovered in this region; all as singletons with low mutual information scores (0.027 to 0.031). Whilst it was hoped that this approach may have discovered additional motifs, it was recognised that the efficiency of the search algorithm in discovering motifs is dependent on the total sequences available for analysis. The use of windows located further upstream of the open reading frame will increase the likelihood over overlap with an adjacent open reading frame, thus limiting the total amount of sequences captured for such an analysis.

**Figure 2:**
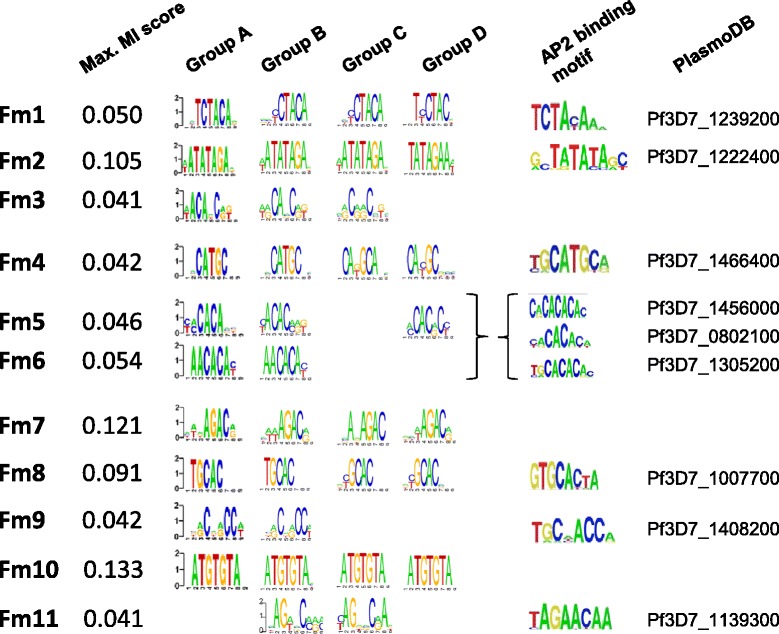
**FIRE motifs (Fm1–11) – a triaged list of putative** ***cis*** **-regulatory elements.** Eleven DNA motifs were repeatedly discovered from searches of sequences groups A to D. The triaged list consist of those motifs which were found in two or more groups of sequences search and met a minimum threshold of 0.04 for the mutual information score. The WebLogo of each discovered motif is represented under the group of sequences in which it was found. To the right, the WebLogo motif of the binding site for a potential cognate AP2 transcription factor is indicated along with the PlasmoDB reference code for the gene that encodes it. Note, the redundancy between Fm5 and Fm6 and those of the indicated cognate AP2 do not allow unambiguous allocation.

This outcome supports the approach adopted here in using repeated rediscovery of DNA motifs; those DNA motifs that are repeatedly discovered have a higher mutual information score. Taking the distribution of mutual information scores in the singleton DNA motifs (0.030 ± 0.004), a cut-off of 0.04 was established for the mutual information score for DNA motifs to be taken forward here. Thus, a short-list of 11 FIRE motifs (Fm1–11) was created, each motif being found in at least two of groups A to D as well as in the original FIRE analysis (Figure 2).

### Fm1-11: a network of cis-acting motifs regulating ring-stage expression in *P. falciparum*?

To explore whether Fm1–11 represent likely *cis*-acting regulatory motifs, they were compared to consensus high affinity DNA binding motifs determined for the *P. falciparum* AP2 specific transcription factors [24,32]. Comparison of these AP2 DNA binding motifs against those of Fm1–11 revealed that six of these (Fm1, 2, 4, 8, 9 and 11) could be unambiguously attributed to a specific AP2 protein. Two further Fm (Fm5 and 6), sharing a degenerate CACA sequence, could not be attributed to a single AP2 transcription factor; instead a cluster of three AP2 transcription factors sharing affinity for these motifs were identified. Thus, of the 11 Fm identified here, eight appear to have a cognate specific transcription factor(s) available to bind them. Intriguingly, for the two Fm (Fm7 and 10) with the highest mutual information scores we could not identify a cognate AP *trans*-acting factor. These two motifs, therefore, may represent either *cis*-acting sites for non-AP2 transcription factors or other factors within the RNA polymerase II complex. Of note is that no other DNA motif identified from the analysis of groups A to D, or from the original FIRE study, had a reliably identifiable cognate AP2 binding partner.

Ranking Fm1–11 by the time during intraerythrocytic development their over-representation correlates with the peak of mRNA accumulation identifies an interesting common temporal property. Fm1–11 are overrepresented in the 5′ flanking sequence of genes that share a peak of mRNA accumulation within the first 24 hours of intraerythrocytic development – correlating with the ring and early trophozoite morphological stages. This contrasts with nuclear transcription run-on data that indicates that overall transcriptional activity during intraerythrocytic development is low during the first third of the cycle (ring stages) [18]. This then increases gradually as the parasites mature in trophozoites and peaks in mid-schizont stages – some 12–16 hours past the latest timepoint linked with Fm1–11. Two additional global processes that also contribute to nucleic acid metabolism also appear to be at play later during intraerythrocytic development. First, mRNA half-life increase as intraerythrocytic development progresses; ranging from a mean of 9.5 minutes in ring stage parasites to 65 minutes in mature schizonts [17]. Second, nucleosome occupancy over intergenic regions is most compact in ring and late-schizont stage parasites [12,13,15,16,19]. The lowest level of nucleosome occupancy is in the mature trophozoite stages, and presumably reflects an increased accessibility of the genomic DNA for nucleic acid metabolism, i.e. transcription and replication. These global mechanisms would appear to provide a reasonable explanation for the characteristic temporal transcriptional upregulation of hundreds of genes during the later stages of intraerythrocytic development. They do not, however, provide a clear insight into the temporal control of gene expression during stages. Our data, to this point, would lead us to suggest that Fm1–11 are *cis*-acting factors that play a role in the promoter-based control of genes expressed during the first 24 hours of intraerythocytic development.

**Figure 3:**
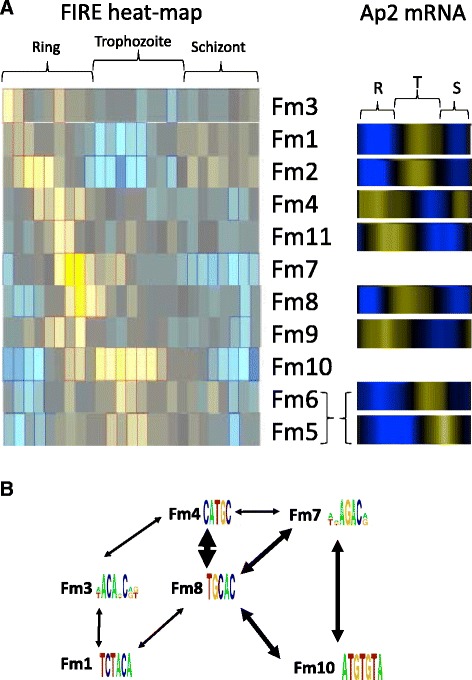
**Temporal distribution of FIRE motif heat-maps and interaction networks for FM1–11. (A)** The motif heat-maps for Fm1–11 are listed according to the earliest point during intraerythrocytic development the associated DNA motif is over-represented. Where a cognate AP2 *trans*-acting factor is suggested, a heat map illustrating the stage-specific accumulation of mRNA for the AP2 during intraerythrocytic development is shown to the right [32]. Yellow and blue colouring represents temporal patterns of up- and down-regulation of mRNA accumulation during intraerythrocytic development. **(B)** An interaction network of colocalized Fm DNA motifs. Colocalization of Fm1–11 in the same 5′ flanking region was determined from the motif interaction heat map produced for each group of sequences searched. Repeated discover of these interactions (i.e. in at least two groups) is represented on this network, with the increasing thickness of the connecting arrows representing discovery in two, three or four searches.

Our last observation relevant to this evolving model of *cis-trans* promoter based control of ring-stage expression comes from a second output file from the FIRE algorithm - a motif interaction heat map. This colour-map illustrates any co-localization of the identified motifs within the same 5′ flanking region. Taking Fm1–11, we determined whether we could repeatedly find the same co-localization of these motifs in each of the searches we performed. Thus, by excluding co-localization of Fm1–11 that occur in only one search of groups A to D, we developed a qualitative network of interactions that were repeatedly discovered between Fm1–11 in two or more searches. This is illustrated in Figure 3 where the thickness of the line emphasizes how often the co-localization was discovered. The strongest link in the network was between Fm4 and Fm8, which was found in all four groups analysed. Interestingly, the AP2 transcription factors associated with Fm4 and Fm8 both share the same ring-stage profile of expression as do the flanking regions of genes in which these motifs are over-represented. This suggests that binding of AP2 to Fm4 and Fm8 may function as positive regulators in the upregulation of transcription of these genes within the ring-stage parasite. As a contrasting observation, expression of the cognate AP2 partner for motifs Fm1, 2, 5 and 6 (as determined from transcriptional and proteomic profiles) is actually upregulated in mature trophozoite stages. This observation could be rationalised if we consider AP2 binding to these Fm motifs acts as a negative regulator of gene expression. That is, a corollary of ring-stage specific expression is that these genes are not subject to global mechanisms that upregulate gene expression in the mature trophozoite stages – thus, AP2 binding to these Fm DNA motifs may act as an isolating negative regulator in maintaining the developmentally-linked expression pattern for these genes. Of note, however, is that whilst the potential for negative regulation though *cis-trans* promoter interactions has been suggested from promotor deletion studies, no direct demonstration for such a role for AP2 has been demonstrated thus far [40,41].

A second interesting feature of this interaction network is the triad of Fm7, 8 and 10. Found in searches of three of the four groups, these Fm represent the highest scoring motifs by mutual information score. Disappointingly, without a cognate AP2 binding partner for Fm 7 and 10, any further discussion relating to a role in directing stage-specific patterns of expression could not be made. However, as a whole, the evidence presented here for a network of colocalised cis-acting motifs, with cognate partners that may act in a positive and negative regulatory role, resonates with a model of combinatorial gene control originally proposed by van Noort and Huynen [42]. They hypothesised that in the apparent absence of a large number of well-defined specific transcription factors in *P. falciparum*, that the necessary complexity necessary to drive the observed cascade of temporally-linked mRNA accumulation could be provided through the combination of a smaller number of transcription factors. The description of such a small number of specific transcription factors, the AP2 family, occurred subsequent to their report in 2006. Importantly, key elements of their model are indicated here, specifically; evidence for multiple *cis*-*trans* interactions within the same 5′ flanking region which may positively or negatively regulate promoter function. A refinement we suggest here is that this molecular mechanism would appear to be particularly important during the first 24 hours of intraerythrocytic development. Subsequent work that indicates multiple binding affinities for AP2 domains, the presence of multiple AP2 domains within a single protein and the potential for AP2 heterodimers suggest that there are additional layers of complexity to explore in these *cis-trans* interactions [23,32,43].

The occurrence and position of eight of the Fm motifs (Fm1, 2, 4–6, 8, 9 and 11) in all *P. falciparum* 5′ flanking regions has been previously mapped and reported (Additional file 1 for [32]). The frequency of Fm motif incidence per gene varies (0.2 to 196.7), but provides some one million occurrences in total over these intergenic regions. As such, it would appear that context, both in terms of position relative to a transcription start site and availability (accounted in part through nucleosome occupancy) to interact with a cognate AP2 partner is important in determining whether a mapped motif actually acts as a *cis*-acting regulatory site. Although transcription start sites have been bioinformatically predicted in *P. falciparum*, few sites have been confirmed experimentally [44]. Recent work, using an improved directional, amplification free, RNAseq approach should shortly provide these key transcriptional landmarks (Chappell, Rayner and Berriman, *pers comm*.). Access of *trans*-acting factors to the DNA motifs is perhaps less on an issue, with *P. falciparum* intergenic regions being relatively depleted of nucleosomes compared to open reading frames [16,19]. Initial analysis of nucleosome binding over the predicted AP2 binding motifs suggests that some 65–97% of the total of all motifs are nucleosome free at some point during intraerythocytic development [32]. With improved resolution stage-specific nucleosome occupancy maps now available [12,13], determining the temporal availability of Fm motifs, specifically those spatially organised around well mapped transcription start sites, offers an opportunity to test the hypothesis that specific transcription factor interactions with these motifs direct stage-specific transcription early during intraerythrocytic development.

## Conclusions

Here we report a repeated bioinformatics search for over-represented DNA motifs within 5′ flanking intergenic regions of *P. falciparum* that we consider most likely to play a role in the stage-specific regulation of genes during intraerythrocytic development. Our search repeatedly identified 11 high scoring DNA motifs, and, significantly, we could identify a likely cognate AP2 *trans*-acting partner for 8 of these. Evidence of preference for regulation of mRNA accumulation during ring-stage development as well as an apparent interaction network between several of these motifs has led us to propose a nuanced modification to the combinatorial gene control model originally proposed by van Noort and Huynen [40]. We propose that that *cis-trans* control of promoter function appears to offer a model for stage-specific expression during ring-stage development, complementing more global mechanisms regulating gene expression during the latter stages of intraerythrocytic development.

## Additional file

Additional file 1:
**FIRE motif heat maps and FIRE interaction heat maps for groups A to D of** ***P. falciparum*** **5′ gene flanking regions.** Full lists of FIRE Weblogo motifs for groups A to D, as well as the original Elemento *et al.* [33] study are shown.

## Abbreviations

Ap2: Apetela-2 domain transcription factor
FIRE: Finding Informative Regulatory Elements
Fm: FIRE motif
MI: Mutual information
