# Supplementary material for: Triaging informative cis-regulatory elements for the combinatorial control of temporal gene expression during Plasmodium falciparum intraerythrocytic development
Source: Parasit Vectors. 2015 Feb 5;8:81. doi: 10.1186/s13071-015-0701-0 (PMC4322800; doi:10.1186/s13071-015-0701-0)
Supplement: Additional file 1: — FIRE motif heat maps and FIRE interaction heat maps for groups A to D of P. falciparum 5′ gene flanking regions. Full lists of FIRE Weblogo motifs for groups A to D, as well as the original Elemento et al. [33] study are shown. [file 13071_2015_701_MOESM1_ESM.pdf]

Supplementary materials:

Triaging informative *cis*-regulatory elements for the combinatorial control of temporal gene expression during *Plasmodium falciparum* intraerythrocytic development

Karen Russell, Richard Emes and Paul Horrocks

Corresponding author: Paul Horrocks. Institute for Science and Technology in Medicine, Keele University, Staffordshire ST5 5BG, United Kingdom. Tel: +44 (0)1782 733670

The FIRE motif heat maps and FIRE interaction heat maps generated from searches of Groups A to D follow. For the FIRE motif maps, each DNA motif is allocated a unique code (eg. A1 for the first motif from group A). The final page correlates the WebLogo motifs from these FIRE motif heat maps to illustrate those that were discovered multiple times across groups A to D, as well as in the original Element0 *et al* (2007) study.

Group A: FIRE motif heat-map

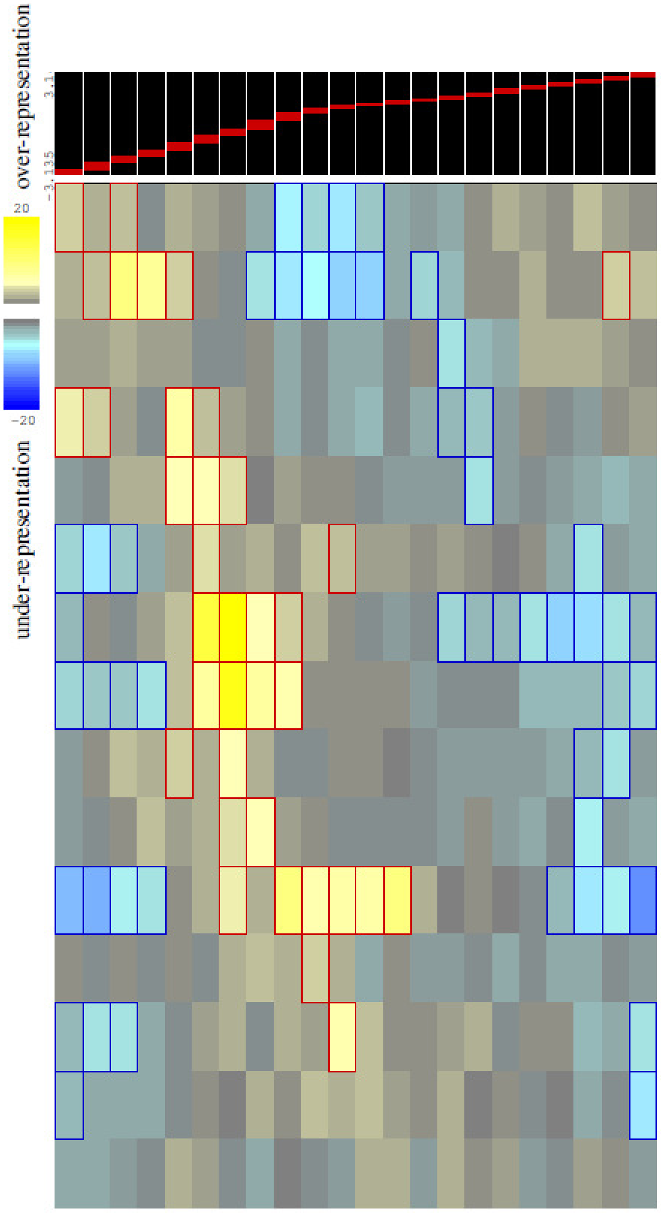

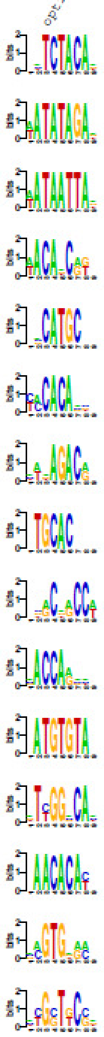

	location	MI (bits)	z-score	robustness	position bias	orientation bias	conservation index	seed
A1	5′	0.050	20.5	10/10	–	–	0.99	TTCTACA
A2	5′	0.092	40.4	10/10	–	–	1.00	ATATAGA
A3	5′	0.028	9.9	10/10	–	–	1.00	ATAATTA
A4	5′	0.041	16.2	10/10	–	–	0.97	ACAACAT
A5	5′	0.040	15.1	10/10	–	–	0.98	ACATGCA
A6	5′	0.046	18.1	10/10	–	–	1.00	ACACAAT
A7	5′	0.116	51.7	10/10	Y	–	1.00	AAAGACA
A8	5′	0.086	36.9	10/10	–	–	1.00	TGCACAC
A9	5′	0.037	13.9	10/10	–	–	0.94	TACAACC
A10	5′	0.031	11.2	9/10	–	←	0.77	ACCAAAA
A11	5′	0.133	59.3	10/10	Y	–	1.00	ATGTGTA
A12	5′	0.026	8.3	7/10	–	←	0.61	TTGGACA
A13	5′	0.044	17.5	10/10	–	–	0.98	AACACAT
A14	5′	0.032	12.0	10/10	–	–	0.98	AGTGTAA
A15	5′	0.024	7.5	6/10	–	–	0.51	TGCTTCC

Group A: FIRE interaction heat map

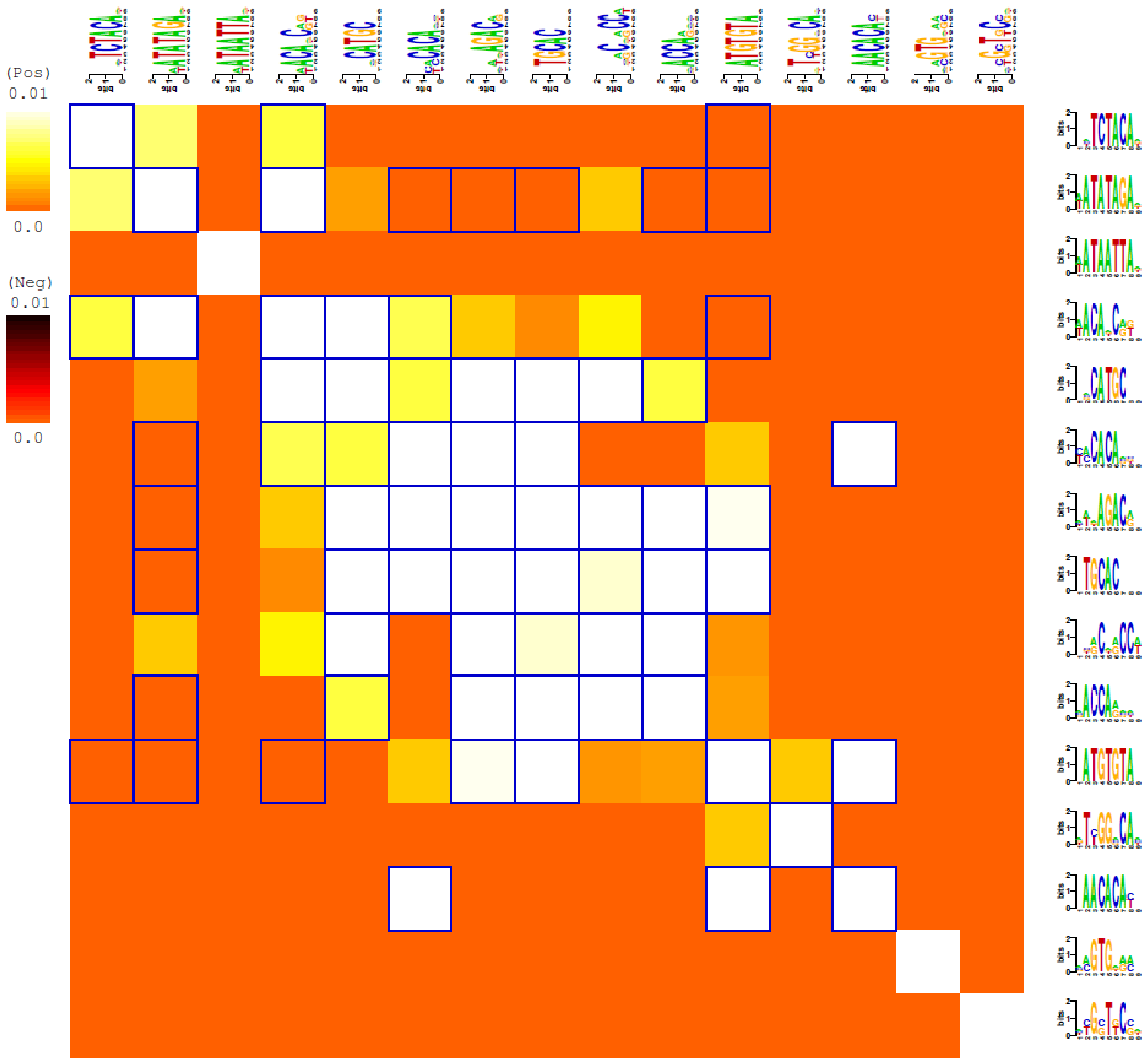

Group B: FIRE motif heat-map

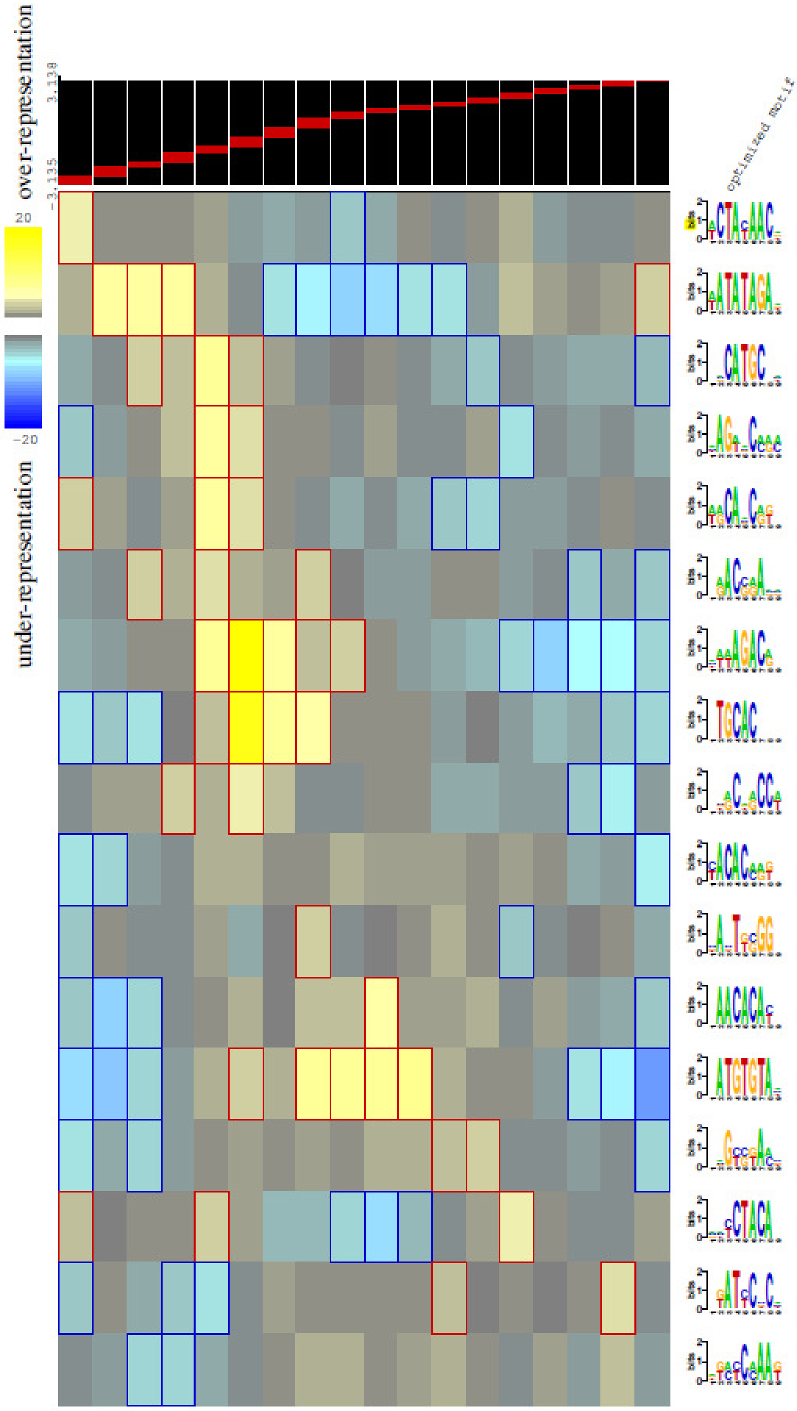

	optimized motif	location	MI (bits)	z-score	robustness	position bias	orientation bias	conservation index	seed
B1		5'	0.029	9.0	6/10	-	←	0.62	CTATAAC
B2		5'	0.105	42.1	10/10	-	-	1.00	ATATAGA
B3		5'	0.042	15.2	10/10	-	-	0.97	ACATGCA
B4		5'	0.041	14.6	9/10	-	←	0.92	AGAACAA
B5		5'	0.041	14.5	10/10	Y	-	1.00	ACAACAT
B6		5'	0.037	12.9	9/10	-	-	0.86	AACCAAG
B7		5'	0.121	48.9	10/10	-	-	1.00	AAAGACA
B8		5'	0.091	35.6	10/10	-	-	1.00	TGCACAC
B9		5'	0.042	14.8	10/10	-	-	0.94	TACAACC
B10		5'	0.037	13.1	10/10	-	-	0.92	ACACAAT
B11		5'	0.028	8.9	6/10	-	→	0.64	ATTTGGG
B12		5'	0.054	20.1	10/10	-	-	0.98	AACACAT
B13		5'	0.132	53.6	10/10	Y	-	1.00	ATGTGTA
B14		5'	0.039	13.7	9/10	-	-	0.94	AGTGTAA
B15		5'	0.046	16.8	10/10	-	-	0.98	TTCTACA
B16		5'	0.030	10.1	8/10	-	-	0.98	TATTCCC
B17		5'	0.029	9.6	10/10	-	←	0.75	TATCAAA

Group B:
FIRE interaction heat-map

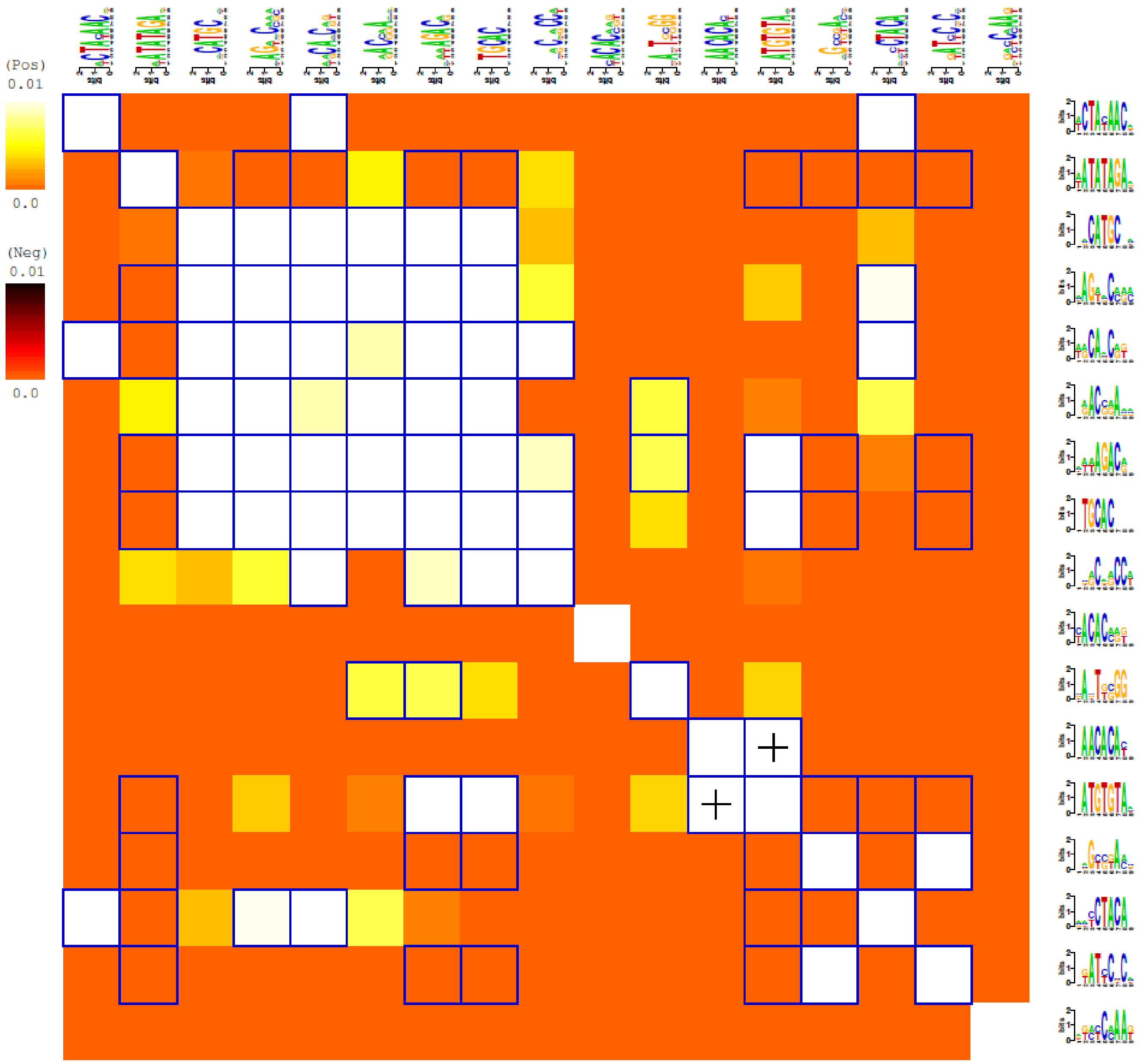

Group C: FIRE motif heat-map

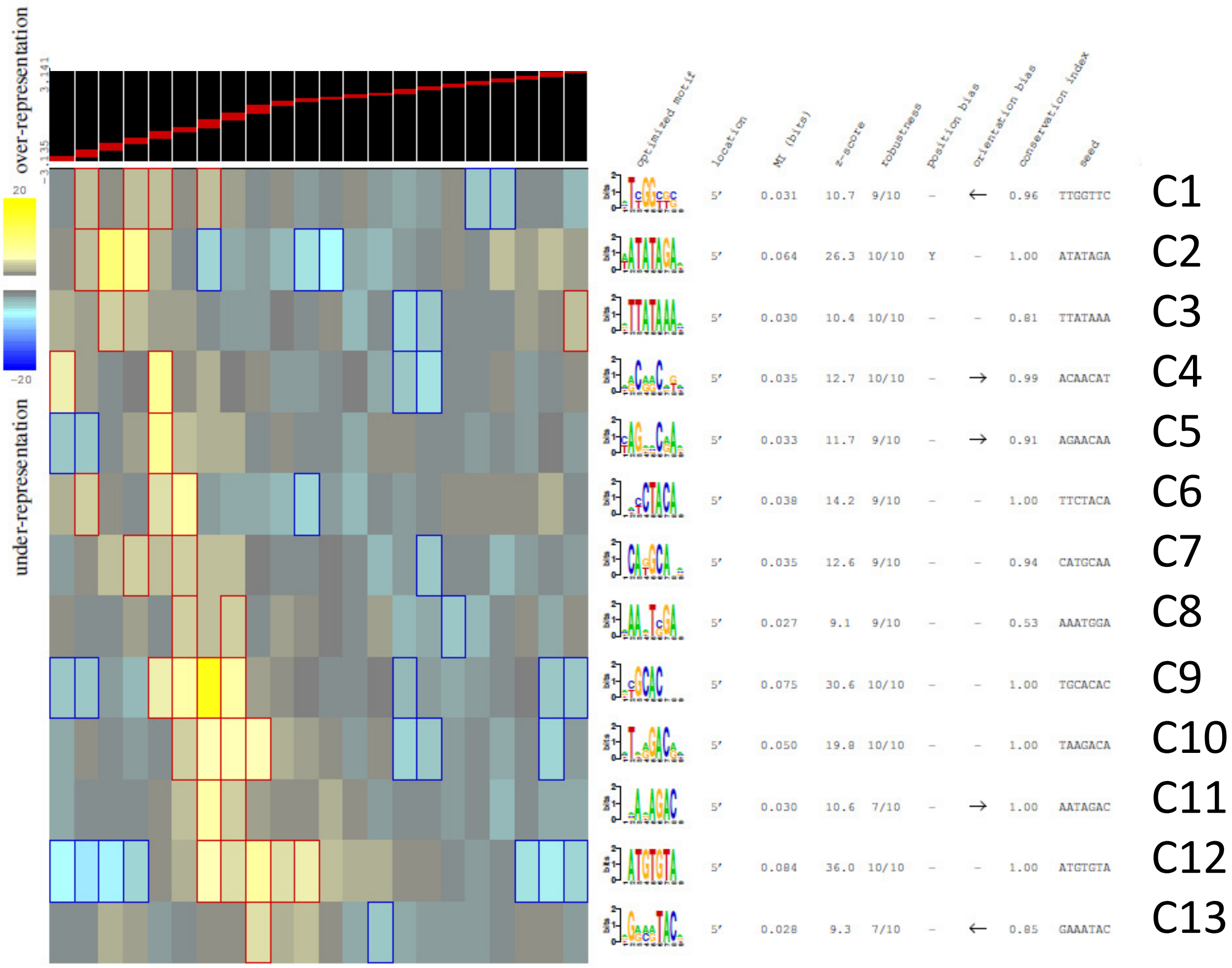

Group C

FIRE interaction heat-map

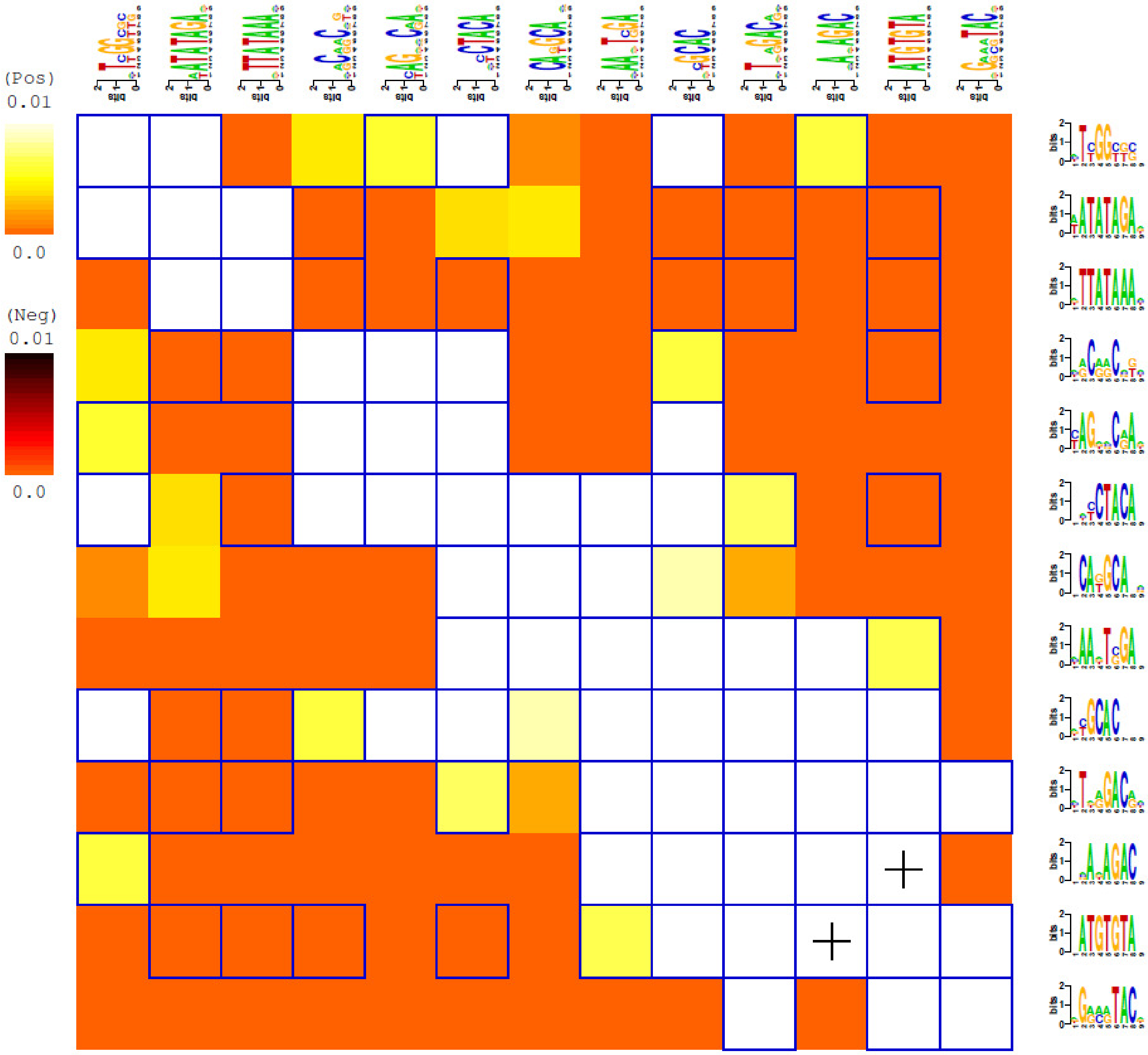

Group D: FIRE motif heat-map

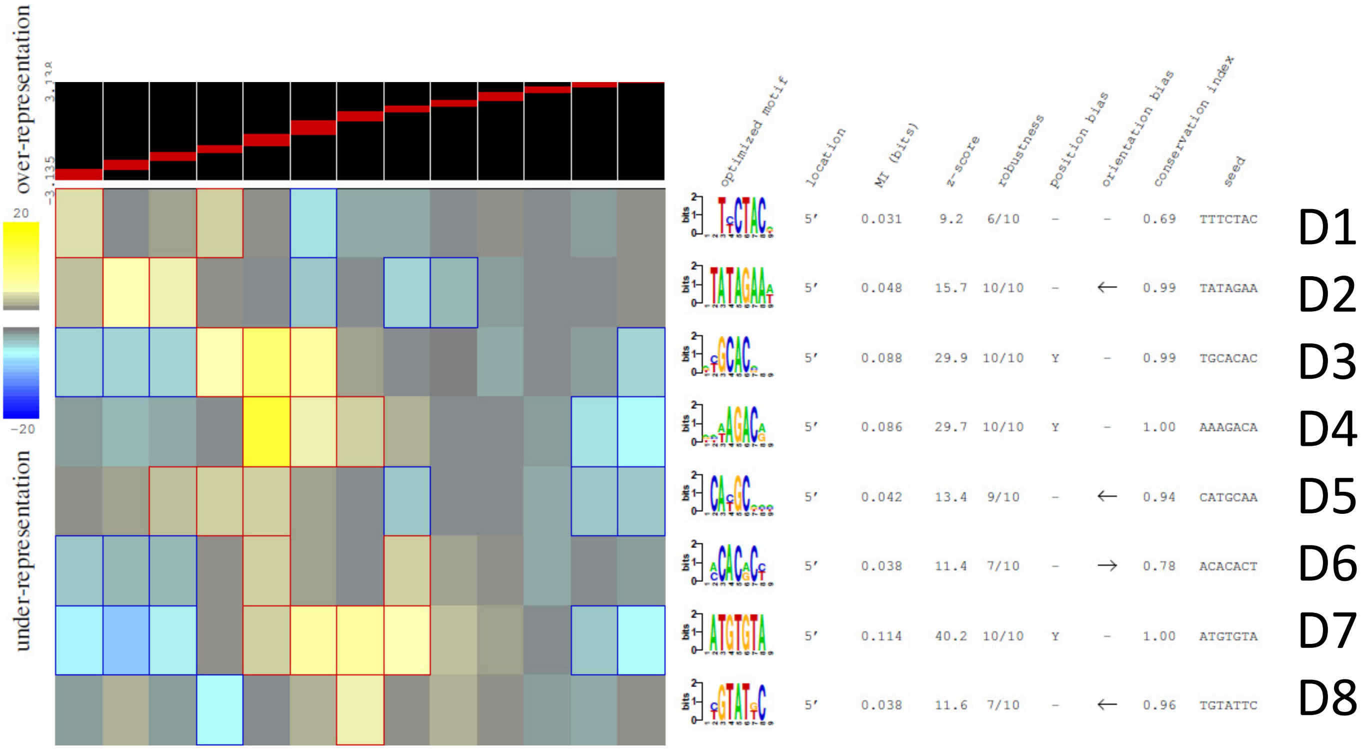

Group D: FIRE interaction heat-map

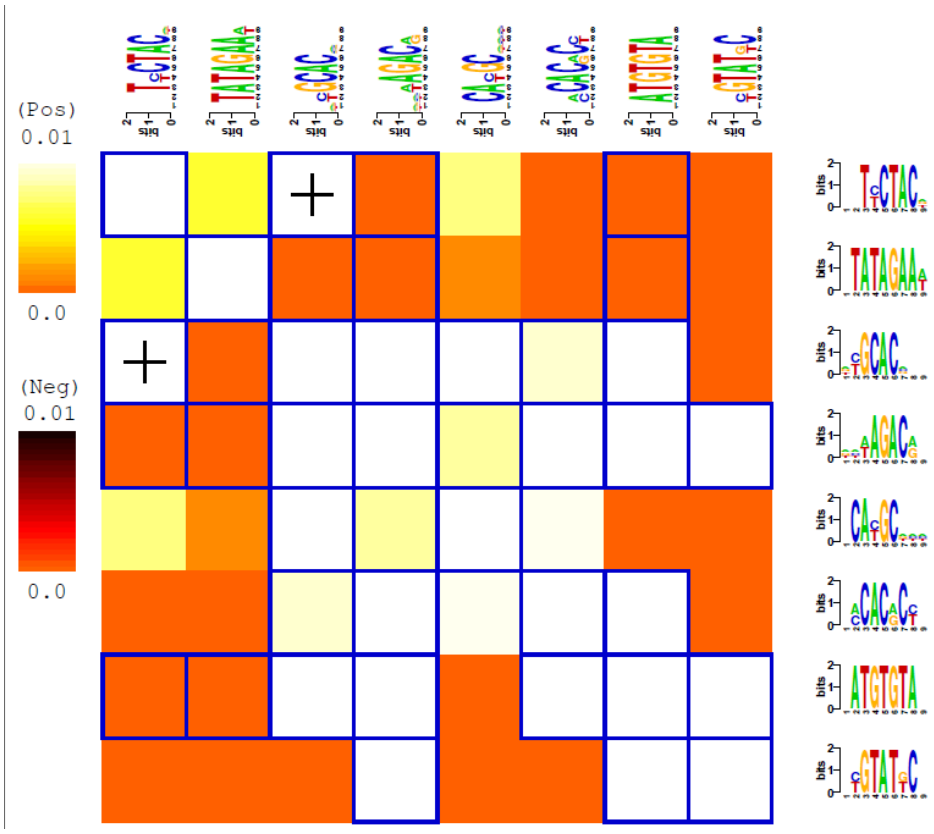

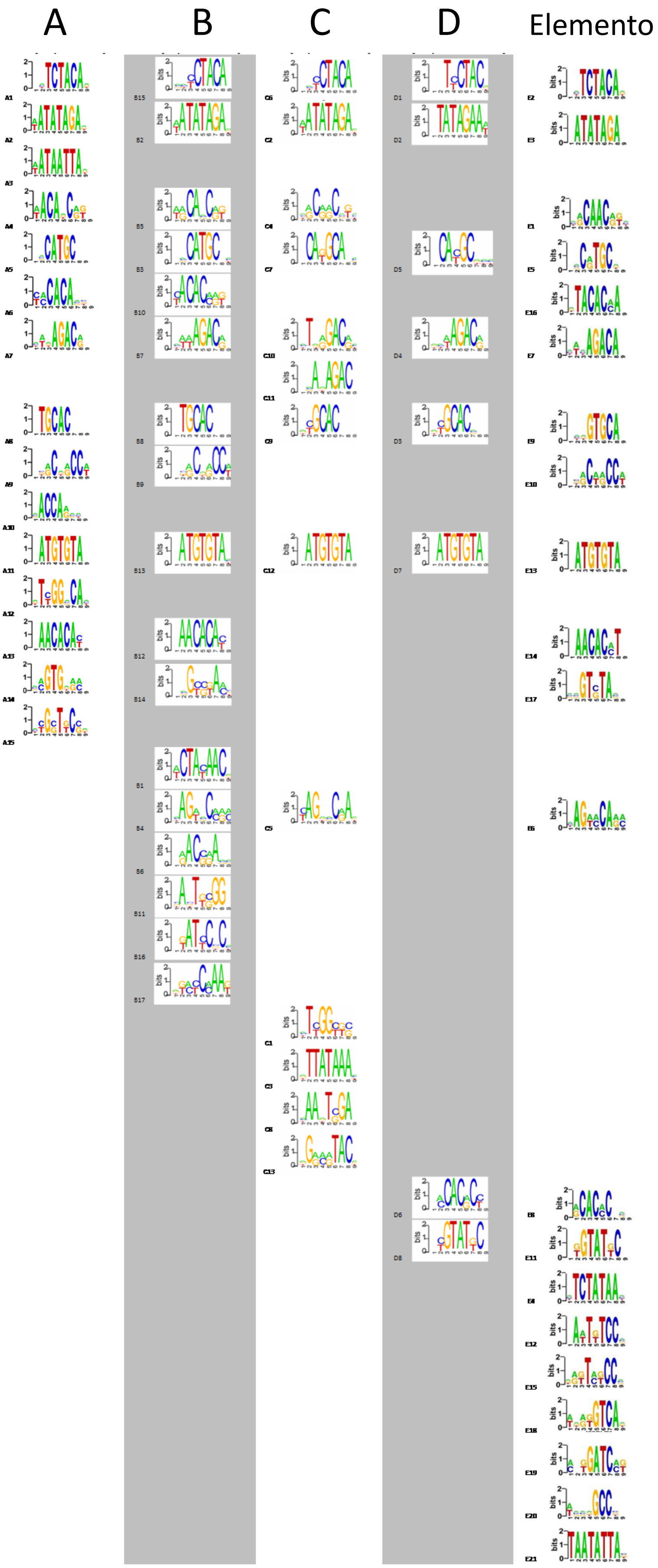
A
B
C
D
Elemento
